# Serum FGF21 Levels Predict the MACE in Patients With Myocardial Infarction After Coronary Artery Bypass Graft Surgery

**DOI:** 10.3389/fcvm.2022.850517

**Published:** 2022-04-06

**Authors:** Wei Xie, Dan Li, Yaru Shi, Ning Yu, Yu Yan, Yingchao Zhang, Qiongli Yu, Yulin Li, Jie Du, Zhuofeng Lin, Fan Wu

**Affiliations:** ^1^School of Pharmaceutical College, Wenzhou Medical University, Wenzhou, China; ^2^The 2nd Affiliated Hospital and Yuying Children's Hospital of Wenzhou Medical University, Wenzhou, China; ^3^Beijing Anzhen Hospital of Capital Medical University, Beijing, China; ^4^Beijing Institute of Heart Lung and Blood Vessel Diseases, Beijing, China

**Keywords:** myocardial infarction, fibroblast growth factor 21 (FGF21), major adverse clinical event (MACE), CABG—coronary artery bypass graft, prognosis value

## Abstract

**Objectives:**

Prognosis evaluation in myocardial infarction (MI) patients with major adverse clinical events (MACE) who have undergone coronary artery bypass graft (CABG) is greatly important to identify high-risk patients. Elevated metabolic hormone fibroblast growth factor 21 (FGF21) is associated with the risk of MI. The aim of this study is to assess the relationship between FGF21 and the incidence of MACE in patients with MI after CABG surgery.

**Methods:**

Patients with three-vessel disease who were scheduled for first-time isolated CABG were enrolled in this project and underwent to evaluate the incidence of MACE during 48 h after CABG surgery, as well as to collect serum samples for FGF21 levels in both preoperative- and postoperative-CABG (pre-CABG and post-CABG).

**Results:**

A total of 265 patients with MI undergoing CABG were enrolled in this study, 21 patients experienced MACE during the 48 h after CAGB surgery. Serum FGF21 levels of patients with MACE at post-CABG were significantly higher than that in patients without MACE [553.7 (433.6) vs. 291.7 (334.4), *p* < 0.001]. Furthermore, among 81 individuals of these 265 patients, a lower level of FGF21 in preoperative-CABG (pre-CABG) and a higher level of FGF21 at postoperative-CABG (post-CABG) were observed in MI patients with MACE as compared to those without MACE respectively [ (275.0 (260.4) vs. 410.3 (420.7), *p* = 0.049; 550.7 (519.9) vs. 370.6 (441.2), *p* = 0.031]. In addition, serum FGF21 levels of MI patients with MACE at post-CABG were significantly increased compared with the baseline levels in pre-CABG [550.7 (519.9) vs.275.0 (260.4) *p* < 0.001]. However, these profiles were not observed in patients without MACE [410.3 (420.7) vs. 370.6 (441.2), *p*=0.2137]. Logistic regression analysis demonstrated that both serum FGF21 and CK-MB levels at post-CABG were independently associated with the incidence of MACE in patients with MI after CABG surgery. Finally, ROC analysis for FGF21 levels of 265 MI patients at post-CABG identified 455.4 pg/ml as an optimal cut-off value to predict MACE, with a sensitivity and specificity of 91.7 and 68.4% respectively.

**Conclusion:**

Serum FGF21 levels at post-CABG are independently associated with the incidence of MACE in patients with MI who have undergone CABG. Measurement of FGF21 may help distinguish patients with MI at a high risk of MACE after CABG surgery.

## Introduction

Ischemic cardiovascular disease remains the leading cause of global morbidity with acute myocardial infarction (AMI) affecting more than 10 million patients worldwide (1). With advancements and implementation of reperfusion therapies such as percutaneous coronary intervention (PCI) and coronary artery bypass graft (CABG) in patients with AMI, the mortality of these patients has significantly reduced in recent decades (2). Even though PCI is widely used as a revascularization strategy to protect ischemic myocardium and to reduce mortality in patients with AMI (3, 4), more and more clinical data suggested that CABG is an indispensable way to build up revascularization in patients with a failed prior PCI, and/or multivessel disease, particularly in patients with complex CAD and diabetes (5, 6). However, growing evidence from clinical investigation demonstrated that the major adverse clinical events (MACE) including re-infarction, heart failure, stroke, and death, which seriously threatened the life and health of patients, are widely observed in short-term (in-hospital) and/or long-term (out-hospital) after CABG surgery in patients with acute myocardial infarction (7), suggesting that evaluation and predication the incidence of MACE in patients with MI after CABG surgery are greatly clinical value.

Fibroblast growth factor 21 (FGF21) is a metabolic hormone with pleiotropic effects on glucose and lipid metabolism and insulin sensitivity (8, 9). Previous studies indicated that FGF21 plays a key role in mediating the metabolic response to fasting/starvation by enhancing fatty acid oxidation, ketogenesis and inducing growth hormone resistance (10, 11). Furthermore, pharmaceutical or genetic replenishment of FGF21 can significantly counteract obesity and its related metabolic disorders in both rodents and nonhuman primates. Recently, our laboratory results indicated that serum FGF21 levels significantly increase in patients with coronary heart disease and are associated with relevant risk factors including abnormal lipid profiles and glucose metabolism (12). Otherwise, FGF21 presents a protective effect against the development of atherosclerotic plaque in apoE knockout mice by induction of adiponectin and suppression of cholesterol synthesis, respectively (13).

On the other hand, FGF21 improves AMI by enhancement of capillary density in the infarct border zone, and reduction of myocardial apoptosis in the remote zone of myocardium in mice (14), and attenuates diabetes-induced myocardial damage by inhibition of high glucose-induced ROS production and mitigation of inflammatory response (15, 16). However, whether metabolic hormone FGF21 is related to the prognosis and progression of myocardial repairment in patients with MI after CABG surgery remains unclear. In this study, we examined and evaluated the relationship between FGF21 and the MACE of patients with MI after CABG surgery. Our data indicated that serum FGF21 levels at post-CABG are strongly associated with the incidence of MACE in patients with MI after CABG surgery, and measurement of circulating FGF21 levels may be beneficial for evaluating the risk of MACE in these MI patients.

## Materials and Methods

### Subjects and Study Design

All participants of patients with MI were enrolled in the ASBA-CABG study (Registry Number: NCT04107571), which was a prospective clinical trial for identification of potential biomarkers of major adverse clinical events in patients with MI after CABG surgery, which was conducted from January 2019 to January 2020 at the Anzhen Hospital of Capital Medical University (Beijing, China). Patients with three-vessel disease, which was defined as angiographically confirmed stenosis ≥50% in all three main epicardial coronary arteries, with or without the involvement of the left main artery, and scheduled for first-time isolated CABG, were enrolled in this project. Patients with previous heart surgery, left ventricular ejection fraction (LVEF) <30%, chronic kidney disease (CKD), lacking informed consent, or in unstable preoperative condition such as patients receiving continuous infusion of inotropics on the day of surgery, were excluded in the current study. Finally, a total of 265 patients with three-vessel disease were included. No treatment intervention was dictated by the protocol for this study. MACE was defined as the occurrence of one or more of these events including re-infarction, stroke, heart failure, and death during 48 hours after CABG surgery in patients with MI. The diagnostic criterion of hypertension is according to 2017 ACC/AHA/AAPA/ABC/ACPM/AGS/APhA/ASH/ASPC/NMA/ PCNA Guideline for the Prevention, Detection, Evaluation, and Management of High Blood Pressure in Adults, and the diagnostic criterion of dyslipidemia is according to the 2017 guideline for the management of dyslipidemia and prevention of cardiovascular disease by American Association of Clinical Endocrinologists and American College of Endocrinology. Blood samples were collected before preoperative-CABG and 6 h after CABG surgery. Demographics, medical history, and conventional biochemical parameters for all participants were collected by independent clinical research coordinators. The study was conducted in accordance with the Declaration of Helsinki, and the protocol was approved by the Review Board of the Anzhen Hospital of Capital Medical University. All participants agreed and signed the informed consent.

### Biomarker Measurement

Whole blood samples collected from patients with MI in both preoperative-CABG (pre-CABG) and postoperative-CABG (post-CABG) were used to isolate serum samples, and then kept at −80°C for subsequent analysis. Circulating levels of FGF21 were measured by a standard enzyme-linked immunosorbent assay kit (Antibody and Immunoassay Service, HKU, HK). Serum cTnI concentrations were examined by a chemiluminescence assay (Beckman Coulter, Access AccuTnI+3). N-terminal pro-B-type natriuretic peptide (NT-proBNP) levels were measured by the Alere Triage immunoassay using the DxI800 platform (Beckman Coulter Diagnostics, Brea, CA, USA). Biochemic parameters including hypersensitive-c-reactive-protein (hs-CRP), creatinine, and LDH were assayed by routinely laboratory methods, respectively.

### Statistical Analysis

Clinical data are described as the number (percentage), or the median (interquartile range) as appropriate. Shapiro-Wilk test was used to assess the normality of data distribution. Differences in continuous variables between two groups were compared using the Mann-Whitney U test, and the χ^2^ test was used for categorical variables. Univariate and multiple logistic regression analyses were used to determine the relationship between FGF21 and the MACE of patients with MI after CABG surgery. The prognostic value of FGF21 against events was determined with ROC curves. A two-sided *p-*value < 0.05 was considered statistically significant. All statistical analyses were performed using IBM SPSS software version 24.0 (SPSS Inc., Chicago, IL, USA), GraphPad Prism 7.0 (GraphPad software, San Diego, CA, USA), or Medcalc 20.014 (Medcalc software Ltd, Ostend, Belgium).

## Results

### Characteristics of Study Subjects

A total of 265 patients with MI undergoing CABG surgery were included in this project and divided into two groups on basis of patients with or without MACE after CABG surgery (Table 1). Twenty-one patients occurred MACE, and the other 244 patients kept a good rehabilitation status to recover after 48 hours of CABG surgery. There were no significant differences in demographics including age, sex distribution and body mass index (BMI), medical history including previous MI, PCI, angina pectoris, atrial fillibration and stroke, comorbidities including hypertension, hyperlipidemia and diabetes mellitus, as well as echocardiography results including LVEF and higher left ventricular end-diastolic diameter (LVEDD) in these MI patients with or without MACE after 48 hours of CABG surgery. In laboratory results, MI patients with MACE in pre-CABG had a higher CK-MB level (*P* < 0.05), a conventional risk factor of AMI, as compared to those without MACE, but no significant differences in other myocardial infarction-related risk factors including NT-pro-BNP, cTnI, and Myoglobin, as well as relevant biochemical parameters including LDH and creatinine were observed in these subjects with or without MACE (Table 1).

**Table 1:** Clinical characteristics of 265 patients with MI after CABG surgery.

	**MACE** **(***n*** = 21)**	**Non-MACE** **(***n*** = 244)**	***P*** **-value**
**Demographics**			
Age (years)	62 (9)	63 (13)	0.306
BMI (kg/m^2^)	27.0 (3.43)	25.7 (4.64)	0.357
male n, (%)	14 (66.7)	178 (73.0)	0.536
Current/former smoker (%)	14 (66.7)	117 (48.0)	0.111
Current/former drinker (%)	5 (23.8)	62 (25.4)	0.839
**Medical history**			
Previous MI (%)	1 (4.8)	4 (1.6)	0.329
Previous PCI (%)	5 (23.8)	63 (25.8)	0.928
Previous angina pectoris (%)	16 (76.2)	215 (88.1)	0.466
Previous stroke (%)	1 (4.8)	33 (13.5)	0.492
Previous atrial fillibration (%)	0 (0)	12 (4.9)	0.296
**Comorbidities**			
Hypertension (%)	13 (61.9)	170 (69.7)	0.460
Hyperlipidemia (%)	8 (38.1)	132 (54.1)	0.159
Diabetes Mellitus (%)	8 (38.1)	103 (42.2)	0.714
**Drug administration**			
Insulin	8 (38.1)	98 (40.1)	0.852
Other lowing-glucose drugs	5 (23.8)	67 (27.5)	0.718
Statin or other lowing-lipid drugs	19 (90.4)	205 (84.1)	0.432
**Echocardiography**			
LVEF (%)	54.0 (10.0)	55.00 (10.0)	0.749
LVEDD (mm)	47.0 (7.5)	45.0 (8.0)	0.961
**Laboratory results**			
NT-pro-BNP (ng/L)	500.0 (477.5)	356.0 (720.0)	0.544
CK-MB (μg/L)	13.0 (11.9)	6.9 (8.8)	0.028
cTnI (μg/L)	0.18 (0.26)	0.14 (0.31)	0.590
cMyo (ng/ml)	321.0 (304.0)	278.4 (260.5)	0.224
LDH (U/L)	181.0 (103.5)	173.0 (84.0)	0.184
Hs-CRP (mg/L)	27.8 (99.4)	46.4 (87.7)	0.253
Hemoglobin A1C (%)	5.5 (0.75)	5.4 (0.87)	0.291
Creatinine (μmol/L)	66.6 (17.7)	65.8 (23.9)	0.714

*Values are presented as absolute number (%), or median (interquartile range) respectively. BMI, body mass index; MI, myocardial infarction; LVEF, left ventricular ejection fraction; LVEDD, left ventricular end-diastolic diameter; NT-proBNP, N-terminal pro-brain B-type natriuretic peptide; CK-MB, creatine kinase-MB; cTnI, cardiac troponin T; cMyo, cardiac myoglobin; LDH, lactate dehydrogenase; hs-CRP, high-sensitivity C-reactive protein*.

### Serum FGF21 Levels From Pre-CABG to Post-CABG Presents an Opposite Change Manner in MI Patients With or Without MACE

To explore the relationship between FGF21 and the incidence of MACE in patients with MI after CABG surgery, serum samples collected from 81 MI patients in both pre- and post-CABG were used to determine circulating FGF21 levels, myocardial infarction-related risk factors, and other biochemical parameters. As shown in Table 2, cardiac function indexes determined by echocardiography indicated that no significant differences between LVEF and LVEDD in MI patients with MACE were observed between pre- and post-CABG respectively (*p* < 0.05). At the same time, these profiles were also observed in these MI patients without MACE after CABG surgery. Similarly, no significant differences of both LVEF and LVEDD values in pre-CABG or at post-CABG were observed in MI patients with or without MACE, respectively.

**Table 2:** The risk parameters related to myocardial infarction in 81 MI patients with or without MACE who had undergone CABG.

	**MACE (*****n*** = **12)**				**Non-MACE (*****n*** **= 69)**				***P*** **for** **Pre-CABG**	***P*** **for** **Post-CABG**
	**Pre-CABG**	**Post-CABG**	**t/z**	***P***	**Pre-CABG**	**Post-CABG**	**t/z**	***P***		
LVEF (%)	54.3 (24.0)	55.2 (16.5)	−0.533	0.594	54.7 (12.0)	56.4 (11.0)	−1.196	0.732	0.677	0.452
LVEDD (mm)	48.7 (9.00)	47.5 (10.3)	−1.527	0.412	48.2 (7.25)	45.0 (5.0)	−1.692	0.561	0.622	0.352
cTnI (ng/ml)	0.01 (0.02)	0.54 (1.09)	−3.957	0.007	0.01 (0.038)	0.49 (1.43)	−5.260	0.000	0.821	0.946
cMyo (ug/L)	17.0 (18.1)	466.0 (279.0)	−1.342	0.009	18.6 (11.2)	276.0 (245.0)	−3.724	0.000	0.409	0.012
CK-MB mass	1.2 (0.98)	5.7 (7.51)	−3.061	0.002	1.2 (0.75)	5.5 (10.05)	−6.607	0.000	0.705	0.821
HB (g/L)	141.0 (22.5)	102.5 (44.45)	6.191	0.000	138.5 (22.5)	101.0 (28.0)	−6.526	0.000	0.371	0.926
PLT ( ×10^9^/L)	253.0 (108.0)	160.0 (76.25)	−3.061	0.002	218.5 (69.3)	150.0 (59.5)	−6.633	0.000	0.263	0.503
CREA (umol/L)	75.1 (16.6)	72.2 (17.1)	−0.784	0.433	73.6 (24.8)	68.2 (32.6)	−1.189	0.201	0.984	0.677
LDH (U/L)	183.5 (35.3)	180.0 (128.8)	−0.235	0.814	183.0 (36.5)	174.0 (83.0)	−0.107	0.915	0.983	0.976
FGF21 (pg/ml)	275.0 (260.4)	550.7 (519.9)	−4.287	0.000	410.3 (420.7)	370.6 (441.2)	−0.256	0.762	0.027	0.031

Data are presented as median (interquartile range). P < 0.005 (0.05^*^1/10) was considered statistically significant, applying post hoc Bonferroni correction. FGF21, fibroblast growth factor 21; LVEF, left ventricular ejection fraction; LVEDD, left ventricular end-diastolic diameter; cTnI, cardiac troponin I; cMyo, cardiac myoglobin; HB, hemoglobin; PLT, platelet; CREA, creatinine; LDH, lactate dehydrogenase.

On the other hand, MI patients without MACE had a higher serum level of MI-related risk factors including cTnI, Myo, and CK-MB mass, as well as biochemical parameters such as hemoglobin, and platelet at post-CABG than that in pre-CABG, respectively (*p* < 0.05). Similarly, the changed profiles of these parameters between pre- and post-CABG were also observed in MI patients with MACE (Table 2). Overall, these data suggest that these conventional myocardial infarction-related risk factors may be unrelated to the incidence of MACE in patients with MI after CABG surgery.

**Figure 1:**
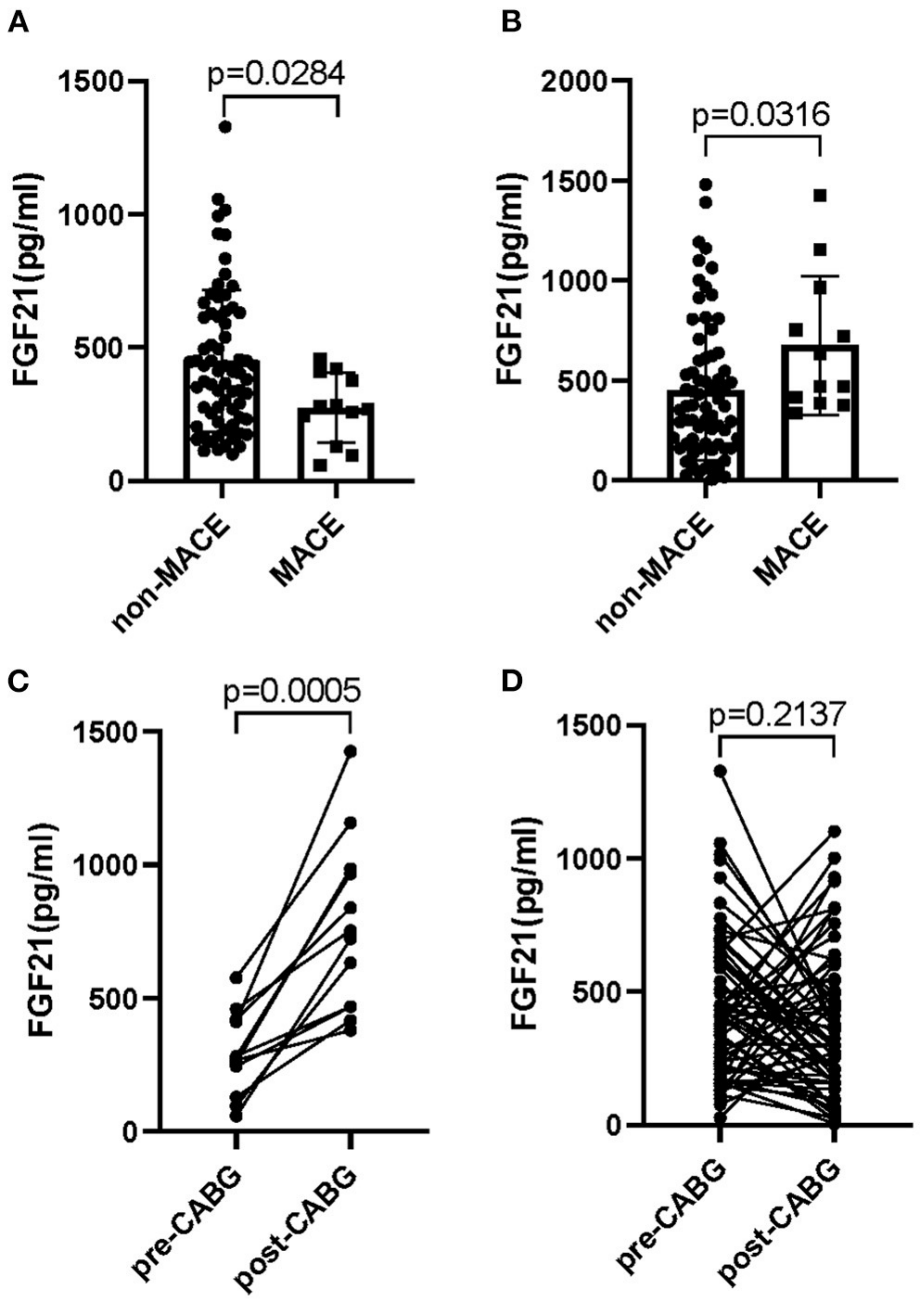
Circulating levels of FGF21 in patients with MI who had undergone CABG. Serum FGF21 levels in pre-CABG **(A)** or post-CABG **(B)** in MI patients with or without MACE; Serum FGF21 levels between pre- and post-CABG in MI patients with MACE **(C)** and without MACE **(D)**.

Different from these conventional risk factors, an interesting result for the change of FGF21 between pre- and post-CABG was observed in patients with MI undergoing CABG surgery. Even though no significant difference of the serum FGF21 levels in 81 patients with MI was observed between pre-CABG ranged from 28.2 to 1327.9 with a median of 380.9 and post-CABG ranged from 125.9 to 3064.3 with a median of 410.0 (*p* = 0.3793). A lower level of FGF21 in pre-CABG and a higher level of FGF21 at post-CABG were observed in MI patients with MACE as compared to those without MACE, respectively (275.0 (260.4) v*s*. 410.3 (420.7), *p* = 0.039; 550.7 (519.9) vs. 370.6 (441.2), *p* = 0.031, Figures 1A,B). Moreover, serum FGF21 levels of MI patients with MACE were significantly increased at post-CABG compared with the baseline levels in pre-CABG (550.7 (519.9) *vs*.275.0 (260.4), *p* = 0.0005, Figure 1C). However, these profiles were not observed in MI patients without MACE (410.3 (420.7) *vs*. 370.6 (441.2), *p* = 0.2137, Figure 1D), even though a slight decrease of serum FGF21 levels was found from baseline to post-CABG. Taken together, these data suggested that the changes of serum FGF21 levels from baseline to post-CABG may be related to the incidence of MACE in patients with MI after CABG surgery.

### Serum FGF21 Levels at Post-CABG Are Strongly Associated With the Incidence of MACE in Patients With MI Undergoing CABG

To further confirm the relationship between FGF21 and the MACE of patients with MI after CABG surgery, we further evaluated the relationship between FGF21 and the MACE in all 265 patients with MI after CABG surgery. Interestingly, serum FGF21 levels of MI patients with MACE at post-CABG were significantly higher than that in MI patients without MACE [553.7 (433.6) vs. 291.7 (334.4), *p* < 0.0012] among these 265 MI individuals. Furthermore, logistic regression analysis also demonstrated that both serum FGF21 and CK-MB levels at post-CABG were significantly associated with the incidence of MACE in these 265 MI patients undergone CAGB after adjusting by age, gender, history of MI, history of drinking, cTnI, smoking, hyperlipidemia, and CK-MB (*p* < 0.05, respectively), suggesting that both serum FGF21 and CK-MB levels at post-CABG are related to the incidence of MACE in patients with MI after CABG surgery (Table 3).

**Table 3:** Logistic regression analysis of MACE in 265 patients with MI who had undergone CABG.

	**Univariate**			**Multivariate^#^**			**Adjustmet**
	**OR**	**95% CI**	***P***	**OR**	**95% CI**	***P***	
Post-FGF21	2.187	1.340~3.570	0.002	2.184	1.317~3.622	0.002	Age, gender, history of MI, history of drink, cTnI, smoking, hyperlipidemia, and CK-MB
Post-CK-MB	1.666	1.080~2.570	0.021	1.609	1.014~2.552	0.044	
Post-LDH	1.249	0.828~1.882	0.289	1.217	0.763~1.941	0.409	
Post-NT-proBNP	1.064	0.714~1.586	0.760	1.061	0.691~1.630	0.786	
Post-cMyO	1.449	0.950~2.211	0.085	1.561	1.067~2.908	0.097	
Post-cTnI	1.164	0.779~1.740	0.458	-	-	-	
Post-HB	1.355	0.896~2.051	0.150	1.250	0.987~2.759	0.176	
Post-PLT	1.207	0.807~1.805	0.360	1.251	0.812~1.925	0.310	
Post-CREA	1.016	0.682~1.513	0.938	1.127	0.700~1.815	0.621	

# Univariate and multivariate logistic regression analysis was performed adjusted by relevant conventional risk factors of myocardial infarction including age, gender, history of MI, history of drink, cTnI, smoking, hyperlipidemia, and CK-MB. P < 0.005 (0.05^*^1/9) was considered statistically significant, applying post hoc Bonferroni correction.

*Post-FGF21, serum FGF21 level at post-CABG; Post-CK-MB, serum creatine kinase-MB levels at post-CABG; Post-LDH, serum lactate dehydrogenase level at post-CABG; Post-NT-proBNP, serum N-terminal pro-brain natriuretic peptide level at post-CABG; Post-cMyO, serum cardiac myoglobin level at post-CABG; Post-cTnI, serum cardiac troponin I level at post-CABG; Post-CRP, serum c-reactive protein level at post-CABG; Post-HB, serum hemoglobin level at post-CABG; Post-PLT, circulating platelet level at post-CABG; Post-CREA, serum creatinine level at post-CABG*.

**Figure 2:**
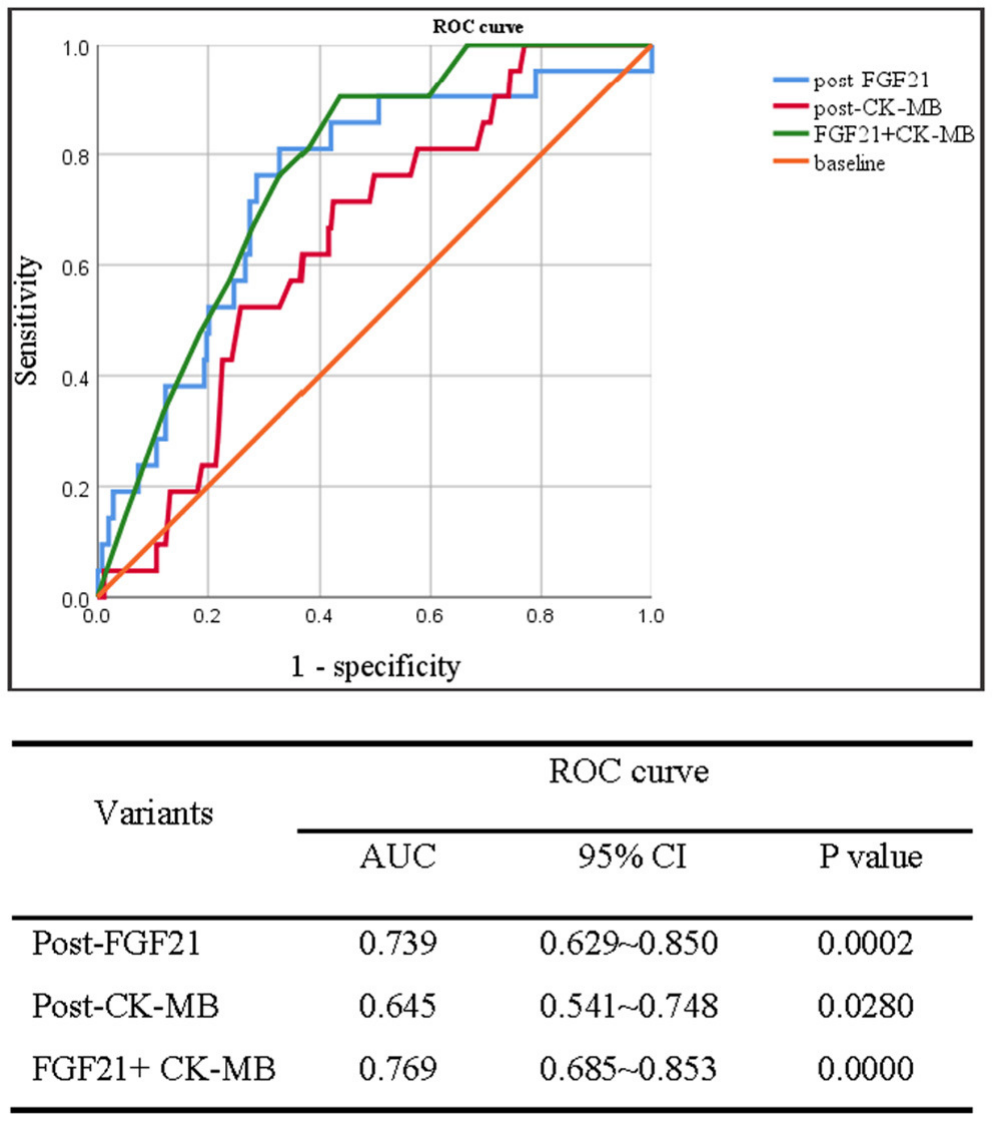
Receiver operating characteristic curve analysis of serum FGF21 levels in between pre- and post-CABG for predicting the MACE of 265 patients with MI after 48 h of CABG surgery. Upper panel: ROC curve; down panel: the area under ROC (AUC) curve.

Data from ROC analysis indicated that the absolute value of AUC for serum FGF21 levels at post-CABG [0.739 (95% CI 0.629–0.850)] was larger than that of CK-MB [0.645 (95% CI 0.541–0.748)], which was considered as a biomarker significantly correlating with late adverse events at 6-months and 1-year post-procedure of PCI (17). Furthermore, a combined analysis for both CK-MB and FGF21 produced a bigger AUC value compared with FGF21 or CK-MB alone (Figure 2). In addition, ROC analysis for FGF21 levels of 265 MI patients at post-CABG identified 455.4 pg/ml as an optimal cut-off value to predict MACE, with a sensitivity and specificity of 91.7 and 68.4%, respectively. Taken together, serum FGF21 levels at post-CABG may be a good predictor for the incidence of MACE in patients with MI after CABG surgery, and measurement of serum FGF21 levels may be beneficial for identifying the high and low risk of MACE in patients with MI who have undergone CABG.

## Discussion

Data from animal-based experiments have demonstrated the protective role of FGF21 in the pathogenesis of MI and myocardial repairment (18, 19), while the clinical relevance of these findings in humans remains poorly characterized. The present study focuses on evaluating the relationships between FGF21 and the incidence of MACE in patients with MI after CABG surgery. Our data provide the first evidence that the increase or decrease of circulating FGF21 levels from the baseline to the post-CABG is related to the incidence of MACE after CABG surgery. Furthermore, serum FGF21 levels at post-CABG, but not in pre-CABG, are independently associated with the MACE in patients with MI after CABG surgery. These findings suggest that FGF21 levels may be a good predictor for identifying the patients with MI at the high risk of MACE from those without MACE after CABG surgery.

Even though PCI and CABG are widely used as a revascularization strategy to protect ischemic myocardium and to reduce mortality in patients with acute MI (3), and CABG is considered as an indispensable way to build up revascularization in patients with a failed prior PCI, and/or multivessel disease, particularly in patients with complex CAD and diabetes (20, 21). However, many MACEs happen in-hospital (short-term) or out-hospital (long-term) including re-infarction, heart failure, stroke, and death, and are frequently observed after CABG surgery, which seriously impacts the prognosis process of patients, even threating the life and health of patients (22, 23). Therefore, predicting the incidence of MACE after CABG surgery and distinguishing patients at the high risk of MACE from these patients with a low risk of MACE are of great clinical value. Recently, imaging results including abnormal LVEF and enlarged ventricular dilation are used to clarify these patients who were recognized as established risk factors for dilated cardiomyopathy (24). Furthermore, many MI-related parameters are promising for distinguishing these patients with a high risk of cardiac damage. For example, NT-pro BNP levels are associated with cardiac damage in relevant cardiovascular disease and are used to predict cardiac death (25). In the current study, our data indicated that a similar change profile of LVEF and LVEDD from pre- to post-CABG is observed in MI patients with or without MACE respectively, suggesting that these imaging data cannot be used to distinguish patients with MI at the high risk of MACE from those without MACE after CABG surgery. Furthermore, MI-related factors including cTnI, Myo, and CK-MB, as well as biochemical parameters such as hemoglobin and platelet, which were partly considered as evaluated biomarkers for myocardial infarction (26), also show a similar manner between pre- and post-CABG in MI patients with or without MACE. These data suggest that these conventional parameters cannot use to clarify the incidence of MACE in patients with MI after CABG surgery, even though some of them are good biomarkers for evaluating the risk of myocardial infarction.

Growing evidence from clinical-based studies has demonstrated that the increased incidences of MACE in patients with MI after CABG surgery seriously influence the prognosis and progression of myocardial repairment, even threatening the life and health of patients (2, 7). Therefore, identification of specific biomarkers for prognosis and risk stratification is very important for clinical assessment and decision-making in patients with MI after CABG surgery. FGF21 is a metabolic hormone with pleiotropic effects on glucose and lipid metabolism and insulin sensitivity (8, 27, 28). Except for the regulation of lipid- and glucose-metabolism in rodents and humans, FGF21 is involved in the pathogenesis of relevant cardiovascular diseases including atherosclerosis, hypertension, coronary heart disease, myocardial infarction, and other myocardial damage such as diabetic cardiomyopathy, dilated cardiomyopathy, and so on (13, 15, 16, 29, 30). Our previous studies indicated that FGF21 is significantly increased in both mouse models of atherosclerosis and patients with coronary heart disease (12, 13). Loss of FGF21 accelerates the plaque formation and the development of atherosclerosis in apoE KO mice, and administration with recombinant human FGF21 protein strongly attenuates the development of atherosclerosis in apoE KO mice by suppression of cholesterol synthesis and production of adiponectin (13, 31). Furthermore, FGF21 attenuates acute myocardial infarction by upregulation of adiponectin in mice (14), and a high circulating FGF21 level is considered as a prognostic biomarker in patients with AMI (32, 33). These data suggest that FGF21 presents an important role in the pathogenesis of acute MI. However, whether FGF21 is related to the prognosis and progression of myocardial repairment in patients with MI after CABG surgery remains unclear. In this study, our primary aim is to evaluate the relationship between FGF21 and the incidences of MACE in MI patients with CABG. As shown in Tables 2, 3, MI patients with MACE have lower serum FGF21 levels at pre-CABG, and patients without MACE have higher serum FGF21 levels. Given that FGF21 attenuates acute myocardial infarction in mice (14), it is possible that lower FGF21 levels increase the risk of MACE in patients with MI after CABG surgery, and measurement of FGF21 has a potential clinical value for distinguishing the high risk of MACE in patients with MI who have undergone CABG.

Recently, more and more clinical evidence has demonstrated that FGF21 is a prognosis biomarker for relevant cardiovascular diseases for its paradoxical upregulation expression during the onset stage of disease (33). We have firstly reported that serum FGF21 levels are significantly increased in patients with CHD and independently associated with adverse lipid profiles (12). Furthermore, serum FGF21 levels are significantly correlated with left ventricular systolic dysfunction and the risks of cardiac death (34). Otherwise, high circulating levels of FGF21 are related to the incidence of re-infarction within 30 days after onset (35). Additionally, serum FGF21 levels are elevated in patients with nonalcoholic fatty liver disease with subclinical atherosclerosis, and baseline level of FGF21 is an independent predictor of atherosclerotic cardiovascular disease and represents a novel biomarker for primary prevention in the general population (36). In this study, our data indicated that serum FGF21 levels at post-CABG, but not pre-CABG, are related to the incidence of MACE in patients with MI after CABG surgery. Otherwise, logistic regression analysis also demonstrated that both serum FGF21 levels at post-CABG surgery are significantly associated with the incidence of MACE in MI patients with CAGB after adjusting by age, gender, history of MI, and cTnI. Moreover, data from ROC analysis indicated that an area under ROC curve (AUC) was 0.694 (95% CI 0.573– 0.815) which indicates the discriminative potential of this value of FGF21 between high and low risk patients. Overall, these data suggest that FGF21 may be a novel predictor for identifying MI patients with MACE at relatively high risk after CABG surgery. The identification of FGF21 and its relationship with the incidence of MACE can discriminate more high-risk patients, who may benefit from early intervention.

## Conclusion

The present study is the first to present the relationship between circulating FGF21 levels and the incidence of MACE in patients with MI after CABG surgery. Serum FGF21 levels at post-CABG may be a specific biomarker to predict the incidence of MACE in patients with MI who have undergone CABG, independent of established conventional risk factors. Serum FGF21 has a strong prognostic value in short-term adverse events of patients with MI after CABG surgery, measurement of FGF21 levels at post-CABG may be beneficial for monitoring the prognosis and progression of myocardial repairment.

## Data Availability Statement

The raw data supporting the conclusions of this article will be made available by the authors, without undue reservation.

## Ethics Statement

The studies involving human participants were reviewed and approved by Review Board of the Anzhen Hospital of Capital Medical University. The patients/participants provided their written informed consent to participate in this study. Written informed consent was obtained from the individual(s) for the publication of any potentially identifiable images or data included in this article.

## Author Contributions

WX and DL have participated in data collection and drafting the manuscript. DL, NY, YZ, and QY performed the statistical analyses. WX, YZ, NY, and YY have performed the laboratory measurements. YL, JD, ZL, and FW have edited and revised the manuscript. All authors have read and approved the final manuscript submitted.

## Funding

This research was supported by China National Funds for Distinguished Young Scientist (No: 81925004 to ZL), National Natural Science Foundation of China (General Program No: 81870317 to ZL).

## Conflict of Interest

The authors declare that the research was conducted in the absence of any commercial or financial relationships that could be construed as a potential conflict of interest.

## Publisher's Note

All claims expressed in this article are solely those of the authors and do not necessarily represent those of their affiliated organizations, or those of the publisher, the editors and the reviewers. Any product that may be evaluated in this article, or claim that may be made by its manufacturer, is not guaranteed or endorsed by the publisher.

## References

[1] Roth GA Mensah GA Johnson CO Addolorato G Ammirati E Baddour LM. Global burden of cardiovascular diseases and risk factors, 1990-2019: update from the GBD 2019 study. J Am Coll Cardiol. (2020) 76:2982–3021. 10.1016/j.jacc.2020.11.010 33309175 PMC7755038

[2] Doenst T Haverich A Serruys P Bonow RO Kappetein P Falk V. PCI and CABG for treating stable coronary artery disease: JACC review topic of the week. J Am Coll Cardiol. (2019) 73:964–76. 10.1016/j.jacc.2018.11.053 30819365

[3] Eagle KA Guyton RA Davidoff R Edwards FH Ewy GA Gardner TJ. ACC/AHA 2004 guideline update for coronary artery bypass graft surgery: a report of the American College of Cardiology/American Heart Association task force on practice guidelines (committee to update the 1999 guidelines for coronary artery bypass graft surgery). Circulation. (2004) 110:1168–76. 10.1161/01.CIR.0000138790.14877.7D 15339866

[4] Algarni KD Maganti M Yau TM. Predictors of low cardiac output syndrome after isolated coronary artery bypass surgery: trends over 20 years. Ann Thorac Surg. (2011) 92:1678–84. 10.1016/j.athoracsur.2011.06.017 21939957

[5] Thielmann M Sharma V Al-Attar N Bulluck H Bisleri G Bunge JJH. ESC joint working groups on cardiovascular surgery and the cellular biology of the heart position paper: perioperative myocardial injury and infarction in patients undergoing coronary artery bypass graft surgery. Eur Heart J. (2017) 38:2392–407. 10.1093/eurheartj/ehx383 28821170 PMC5808635

[6] DeFilippis AP Chapman AR Mills NL de Lemos JA Arbab-Zadeh A Newby LK. Assessment and treatment of patients with type 2 myocardial infarction and acute nonischemic myocardial injury. Circulation. (2019) 140:1661–78. 10.1161/CIRCULATIONAHA.119.040631 31416350 PMC6855329

[7] Goodman SG Aylward PE Szarek M Chumburidze V Bhatt DL Bittner VA. Effects of alirocumab on cardiovascular events after coronary bypass surgery. J Am Coll Cardiol. (2019) 74:1177–86. 10.1016/j.jacc.2019.07.015 31466614

[8] Lin Z Tian H Lam KS Lin S Hoo RC Konishi M. Adiponectin mediates the metabolic effects of FGF21 on glucose homeostasis and insulin sensitivity in mice. Cell Metab. (2013) 17:779–89. 10.1016/j.cmet.2013.04.005 23663741

[9] Holland WL Adams AC Brozinick JT Bui HH Miyauchi Y Kusminski CM. An FGF21-adiponectin-ceramide axis controls energy expenditure and insulin action in mice. Cell Metab. (2013) 17:790–7. 10.1016/j.cmet.2013.03.019 23663742 PMC3667496

[10] Fazeli PK Lun M Kim SM Bredella MA Wright S Zhang Y. FGF21 and the late adaptive response to starvation in humans. J Clin Invest. (2015) 125:4601–11. 10.1172/JCI83349 26529252 PMC4665770

[11] Holmes D. Metabolism: fasting induces FGF21 in humans. Nat Rev Endocrinol. (2016) 12:3. 10.1038/nrendo.2015.202 26585659

[12] Lin Z Wu Z Yin X Liu Y Yan X Lin S. Serum levels of FGF-21 are increased in coronary heart disease patients and are independently associated with adverse lipid profile. PLoS ONE. (2010) 5:e15534. 10.1371/journal.pone.0015534 21206918 PMC3012070

[13] Lin Z Pan X Wu F Ye D Zhang Y Wang Y. Fibroblast growth factor 21 prevents atherosclerosis by suppression of hepatic sterol regulatory element-binding protein-2 and induction of adiponectin in mice. Circulation. (2015) 131:1861–71. 10.1161/CIRCULATIONAHA.115.015308 25794851 PMC4444420

[14] Joki Y Ohashi K Yuasa D Shibata R Ito M Matsuo K. FGF21 attenuates pathological myocardial remodeling following myocardial infarction through the adiponectin-dependent mechanism. Biochem Biophys Res Commun. (2015) 459:124–30. 10.1016/j.bbrc.2015.02.081 25712519

[15] Yang H Feng A Lin S Yu L Lin X Yan X. Fibroblast growth factor-21 prevents diabetic cardiomyopathy via AMPK-mediated antioxidation and lipid-lowering effects in the heart. Cell Death Dis. (2018) 9:227. 10.1038/s41419-018-0307-5 29445083 PMC5833682

[16] Zhang C Huang Z Gu J Yan X Lu X Zhou S. Fibroblast growth factor 21 protects the heart from apoptosis in a diabetic mouse model via extracellular signal-regulated kinase 1/2-dependent signalling pathway. Diabetologia. (2015) 58:1937–48. 10.1007/s00125-015-3630-8 26040473

[17] Javaid A Buch AN Steinberg DH Pinto Slottow T Roy P Pichard AD. Does creatine kinase-MB (CK-MB) isoenzyme elevation following percutaneous coronary intervention with drug-eluting stents impact late clinical outcome? Catheter Cardiovasc Interv. (2007) 70:826–31. 10.1002/ccd.21248 17621656

[18] Domouzoglou EM Naka KK Vlahos AP Papafaklis MI Michalis LK Tsatsoulis A. Fibroblast growth factors in cardiovascular disease: The emerging role of FGF21. Am J Physiol Heart Circ Physiol. (2015) 309:H1029–38. 10.1152/ajpheart.00527.2015 26232236 PMC4747916

[19] Geng L Lam KSL Xu A. The therapeutic potential of FGF21 in metabolic diseases: from bench to clinic. Nat Rev Endocrinol. (2020) 16:654–67. 10.1038/s41574-020-0386-0 32764725

[20] Nystrom T Sartipy U Franzen S Eliasson B Gudbjornsdottir S Miftaraj M. PCI versus CABG in patients with type 1 diabetes and multivessel disease. J Am Coll Cardiol. (2017) 70:1441–51. 10.1016/j.jacc.2017.07.744 28851544

[21] Mehta RH Leimberger JD van Diepen S Meza J Wang A Jankowich R. Levosimendan in patients with left ventricular dysfunction undergoing cardiac surgery. N Engl J Med. (2017) 376:2032–42. 10.1056/NEJMoa1616218 28316276

[22] Gaba P Gersh BJ Ali ZA Moses JW Stone GW. Complete versus incomplete coronary revascularization: definitions, assessment and outcomes. Nat Rev Cardiol. (2021) 18:155–68. 10.1038/s41569-020-00457-5 33067581

[23] Shroyer AL Hattler B Wagner TH Collins JF Baltz JH Quin JA. Five-Year outcomes after on-pump and off-pump coronary-artery bypass. N Engl J Med. (2017) 377:623–32. 10.1056/NEJMoa1614341 28813218

[24] Patel AR Kramer CM. Role of cardiac magnetic resonance in the diagnosis and prognosis of nonischemic cardiomyopathy. JACC Cardiovasc Imaging. (2017) 10:1180–93. 10.1016/j.jcmg.2017.08.005 28982571 PMC5708889

[25] Whitman IR Vittinghoff E DeFilippi CR Gottdiener JS Alonso A Psaty BM. NT -pro BNP as a mediator of the racial difference in incident atrial fibrillation and heart failure. J Am Heart Assoc. (2019) 8:e010868. 10.1161/JAHA.118.010868 30912456 PMC6509704

[26] Fan J Ma J Xia N Sun L Li B Liu H. Clinical value of combined detection of CK-MB, MYO, cTnI and plasma NT-proBNP in diagnosis of acute myocardial infarction. Clin Lab. (2017) 63:427–33. 10.7754/Clin.Lab.2016.160533 28271683

[27] Oishi K Uchida D Ishida N. Circadian expression of FGF21 is induced by PPARalpha activation in the mouse liver. FEBS Lett. (2008) 582:3639–42. 10.1016/j.febslet.2008.09.046 18840432

[28] Zhang X Yeung DC Karpisek M Stejskal D Zhou ZG Liu F. Serum FGF21 levels are increased in obesity and are independently associated with the metabolic syndrome in humans. Diabetes. (2008) 57:1246–53. 10.2337/db07-1476 18252893

[29] Ong KL Januszewski AS O'Connell R Jenkins AJ Xu A Sullivan DR. The relationship of fibroblast growth factor 21 with cardiovascular outcome events in the fenofibrate intervention and event lowering in diabetes study. Diabetologia. (2015) 58:464–73. 10.1007/s00125-014-3458-7 25425220

[30] Pan X Shao Y Wu F Wang Y Xiong R Zheng J. FGF21 prevents angiotensin II-induced hypertension and vascular dysfunction by activation of ACE2/Angiotensin-(1-7) axis in mice. Cell metabolism. (2018) 27:1323–37 e5. 10.1016/j.cmet.2018.04.002 29706566

[31] Jin L Lin Z Xu A. Fibroblast growth factor 21 protects against atherosclerosis via fine-tuning the multiorgan crosstalk. Diabetes Metab J. (2016) 40:22–31. 10.4093/dmj.2016.40.1.22 26912152 PMC4768047

[32] Chen H Lu N Zheng M. A high circulating FGF21 level as a prognostic marker in patients with acute myocardial infarction. Am J Transl Res. (2018) 10:2958–66. 30323882 PMC6176227

[33] Zhang Y Yan J Yang N Qian Z Nie H Yang Z. High-level serum fibroblast growth factor 21 concentration is closely associated with an increased risk of cardiovascular diseases: a systematic review and meta-analysis. Front Cardiovasc Med. (2021) 8:705273. 10.3389/fcvm.2021.705273 34513950 PMC8427036

[34] Shen Y Zhang X Pan X Xu Y Xiong Q Lu Z. Contribution of serum FGF21 level to the identification of left ventricular systolic dysfunction and cardiac death. Cardiovasc Diabetol. (2017) 16:106. 10.1186/s12933-017-0588-5 28821258 PMC5562996

[35] Zhang W Chu S Ding W Wang F. Serum level of fibroblast growth factor 21 is independently associated with acute myocardial infarction. PLoS ONE. (2015) 10:e0129791. 10.1371/journal.pone.0129791 26091256 PMC4474722

[36] Wu L Qian L Zhang L Zhang J Zhou J Li Y. Fibroblast growth factor 21 is related to atherosclerosis independent of nonalcoholic fatty liver disease and predicts atherosclerotic cardiovascular events. J Am Heart Assoc. (2020) 9:e015226. 10.1161/JAHA.119.015226 32431189 PMC7428997

